# Serum procalcitonin levels can be used to differentiate between inflammatory and non-inflammatory diarrhea in acute infectious diarrhea

**DOI:** 10.1097/MD.0000000000011795

**Published:** 2018-08-10

**Authors:** Hae Jin Shin, Sun Hyung Kang, Hee Seok Moon, Jae Kyu Sung, Hyun Yong Jeong, Ju Seok Kim, Jong Seok Joo, Eaum Seok Lee, Seok Hyun Kim, Byung Seok Lee

**Affiliations:** a Division of Gastroenterology, Department of Internal Medicine, Aerospace Medical Center, Republic of Korea Air Force, Cheongwon-gun, Chungcheongbuk-do; b Division of Gastroenterology, Department of Internal Medicine, Chungnam National University School of Medicine, Daejeon, Republic of Korea.

**Keywords:** acute infectious diarrhea, inflammatory diarrhea, non-inflammatory diarrhea, procalcitonin

## Abstract

In this study, we assess the possibility of using procalcitonin levels to differentiate between inflammatory diarrhea and non-inflammatory diarrhea in acute infectious diarrhea.

We reviewed the records of 1176 patients who had symptoms of diarrhea, fever (≥37.8 °C), and abdominal pain between March 2011 and May 2015. After applying exclusion criteria, a sample of 514 patients was considered for study. The patient sample was divided into Group A and Group B for inflammatory diarrhea and non-inflammatory diarrhea, respectively. The assessment involved comparing the laboratory characteristics with the clinical characteristics of the groups.

The characteristics of Group A, such as white blood cell (WBC), C-reactive protein (CRP), absolute neutrophil count (ANC), and procalcitonin levels, were relatively higher than those of Group B (*P* < .001 for Group A). A receiver operator characteristic (ROC) analysis revealed that the highest area-under-the-curve (AUC) value of procalcitonin (0.797; 95% confidence interval [CI] [0.760, 0.831]; *P* < .001), could be used to differentiate between the 2 groups. Procalcitonin exhibited a sensitivity and a specificity of 87.03% and 68.75%, respectively, at a 0.08 ng/mL cut-off level.

Procalcitonin was a good candidate biomarker of inflammatory diarrhea than other inflammatory markers.

## 1 Introduction

From a global perspective, infectious diarrhea is one of the most common diseases, predominantly among the developing nations. The fatality level associated with infectious diarrhea has remained high despite enhanced hygiene and treatment.^[1]^ Acute infectious diarrhea can be categorized as inflammatory (cytotoxin or invasion) and non-inflammatory (enterotoxin).^[2]^ Enterotoxin causes watery diarrhea by acting directly on secretory mechanisms in the intestinal mucosa, while cytotoxin or bacterial invasion cause destruction of mucosal cells and associated inflammatory diarrhea. The wide range of clinical manifestations of acute infectious diarrhea is matched by the wide variety of infectious agents involved, including viruses, bacteria, and parasites. A significant number of non-inflammatory cases of diarrhea are caused by viruses, parasites, and a range of bacteria, with the common treatment being oral hydration and proper nutrition. Inflammatory diarrhea results from invasive pathogens, such as *Entamoeba histolytica*, enteroinvasive *Escherichia coli* (EIEC), *Shigella*, shiga toxin-producing *E coli* (STEC), *Salmonella*, and *Campylobacter*, and is related to immense inflammation in the intestines; a distinctive diagnosis is essential to determine the necessary antimicrobial therapy. This indicates why it is compulsory to distinguish non-inflammatory diarrhea from inflammatory diarrhea upon a patient's admission.^[3]^ Despite the fact that stool culture remains the primary technique employed in differentiating non-inflammatory from inflammatory diarrhea, it is very costly. Furthermore, about 80% of samples cannot be determined using stool culture, and professionals and high-tech equipment are needed to analyze stools.^[3,4]^

The rapid stool examination requiring a microscopic assessment for erythrocytes and leukocytes has limited value for distinguishing between pathogens in watery diarrhea.^[5,6]^ Both the lactoferrin latex agglutination test and the fecal occult blood test (FOBT) are simple, quick, and cost-effective tests that can be used to screen for inflammatory diarrhea. However, these tests require modern laboratories with competent technicians, and they have a low specificity and sensitivity.^[7]^

An increased level of procalcitonin is considered a key laboratory indication of acute infection, and it has been confirmed as a marker of bacterial infection among intensive care unit and post-surgical patients, as well as neonatal sepsis patients.^[8,9]^ As an inactive calcitonin precursor, procalcitonin is a 116-amino acid polypeptide glycoprotein with a 13 kDa molecular weight.^[8]^ It is produced only in the thyroid gland C cells at normal metabolic settings. Serum concentrations in healthy persons are very low, <0.05 ng/mL, or sometimes even undetectable.^[10]^ Increased levels of procalcitonin were initially reported by French authors in patients with sepsis, as well as fungal and bacterial infections.^[11]^ Some of the benefits associated with using procalcitonin as an inflammatory marker is that it is a simple and reliable test that has a quick turnaround time of 2 hours for the results.^[12]^ From a general assessment and meta-analysis perspective, procalcitonin performed better than C-reactive protein (CRP) as a biomarker in relation to the diagnostic precision of bacterial infection among inpatients.^[13]^ The function of procalcitonin in the diagnosis of necrotizing pancreatitis,^[14]^ in the discrimination of infectious and noninfectious causes of early acute respiratory distress syndrome,^[15]^ and in surveying infection in transplant recipients has been studied.^[16]^ There have also been confirmed reports concerning the correlation of procalcitonin with disease activity in a range of autoimmune settings, such as the Wegener granulomatous disease.^[17]^

The objective of the present study centered on examining the efficacy of inflammatory markers, particularly procalcitonin, in the discrimination between inflammatory and non-inflammatory diarrhea in patients with acute infectious diarrhea. The study also examined the significance of such infection markers during antibiotic therapy, since the antibiotic therapy for gastroenteritis is considered highly controversial among stable patients.^[18]^ Avoiding needless antibiotic therapy in healthcare facilities is a top concern among professionals in this sector because of antibiotic resistance and allergic reactions.^[19]^

## 2 Materials and methods

### 2.1 Study population and design

This study employed a retrospective methodology based on data from a tertiary hospital in Daejeon, Republic of Korea. We reviewed the records of 1176 patients who presented at the hospital with symptoms of diarrhea, fever (≥37.8 °C), and abdominal pain between March 2011 and May 2015. The eligibility criteria involved undergoing a colonoscopy or abdominal computed tomography (CT) within the first 3 days of being admitted, as well as sampling blood during admission. The patients were subdivided into Group A and Group B for inflammatory diarrhea and non-inflammatory diarrhea, respectively.

**Figure 1:**
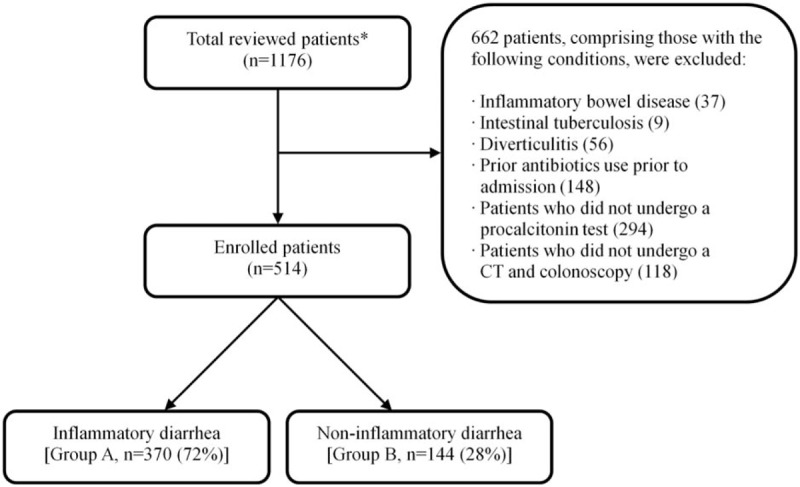
Flow diagram of this study. Asterisks represent the patients presented with diarrhea, fever (≥37.8 °C), and abdominal pain.

The assessment involved comparing the laboratory characteristics with the clinical characteristics of the 2 groups. For Group A, the inflammatory diarrhea group, patients had the following conditions: bowel wall thickening >5 mm,^[20]^ pericolonic stranding or fluid collection at the distal ileum or colon on abdominal CT; and hemorrhage, erythema, edema, or ulcer on colonoscopy. For Group B, the group with non-inflammatory diarrhea, the patients did not show abnormal findings during a colonoscopy or abdominal CT. The exclusion criteria involved patients with inflammatory bowel disease, intestinal tuberculosis, or diverticulitis, patients who had taken antibiotics prior to admission, and patients who did not check abdominal CT, colonoscopy, and serum procalcitonin. From the 1176 records of patients examined, 662 patients were excluded, while 514 patients were included in the study (Fig. 1). For this retrospective study, written informed consent was not required.

### 2.2 Statistical analysis

The statistical software used in analyzing the collected data was SPSS software, version 12.0 (SPSS, Chicago, IL). Categorical data were analyzed with chi-squared statistics or Fisher exact test. Continuous data were analyzed using a *t* test. To establish the independent indicators of inflammatory diarrhea, we performed a multivariate logistic regression analysis with the variables that were significant at 0.05 level through a univariate analysis. A <.05 *P* value signified statistical importance. Both the specificity and the sensitivity were computed by engaging a receiver operator characteristic (ROC) curves analysis.

## 3 Results

From the 514 patients who were considered eligible for this study, 72% (n = 370) were included in the inflammatory diarrhea group (Group A), while the remaining 28% (n = 144) were included in the non-inflammatory diarrhea group (Group B). Tables 1 and 2 present the baseline clinical characteristics and the laboratory characteristics of the 2 groups.

**Table 1:**
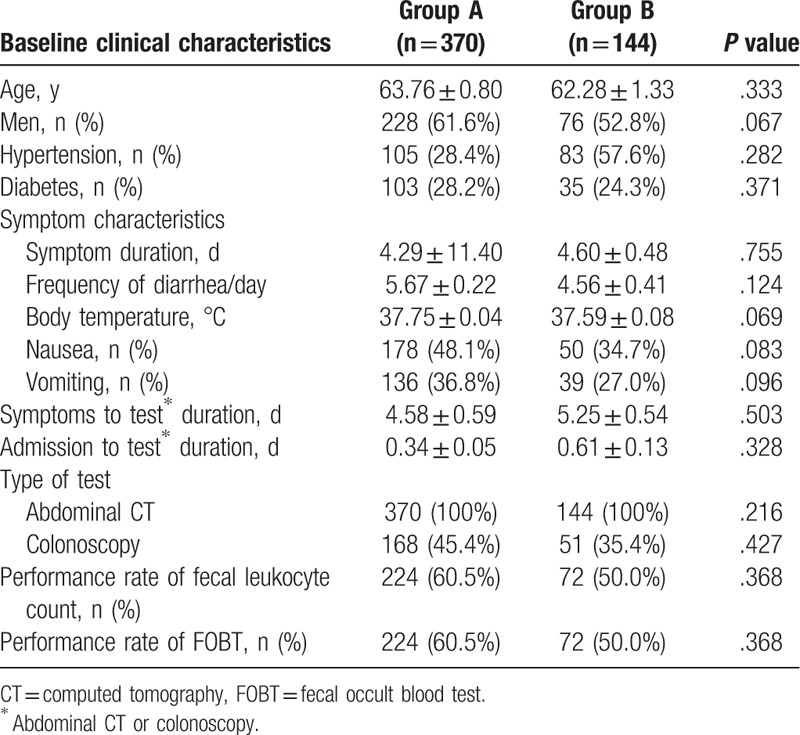
Baseline clinical characteristics of the study cohort.

**Table 2:**
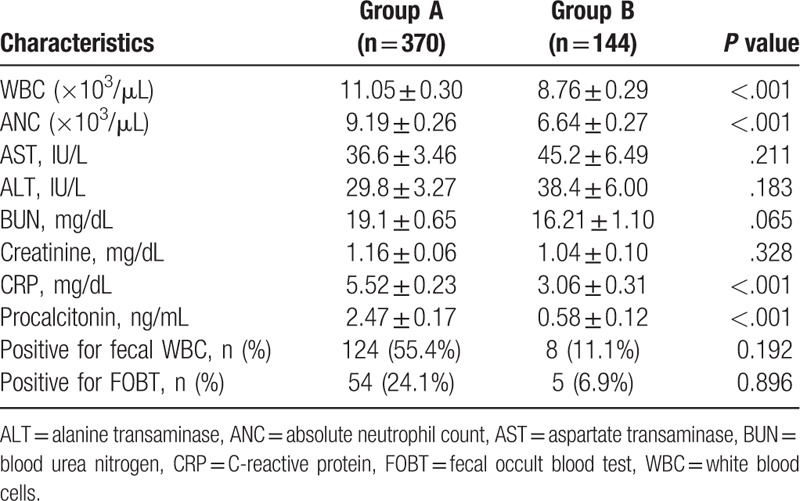
Baseline laboratory results for the study cohort.

The results did not reveal any significant clinical variations between the 2 groups. From the laboratory tests, it was evident that the white blood cell (WBC) count, the absolute neutrophil count (ANC), and the CRP and procalcitonin levels were statistically higher in Group A (*P* < .001) compared with Group B. The analysis of a multivariate logistic regression showed that the noteworthy independent predictors for inflammatory diarrhea were CRP and procalcitonin levels (Table 3).

**Table 3:**
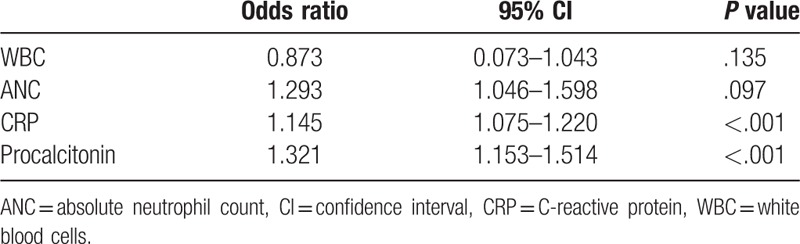
Multivariate analysis of possible risk factors for inflammatory diarrhea.

**Figure 2:**
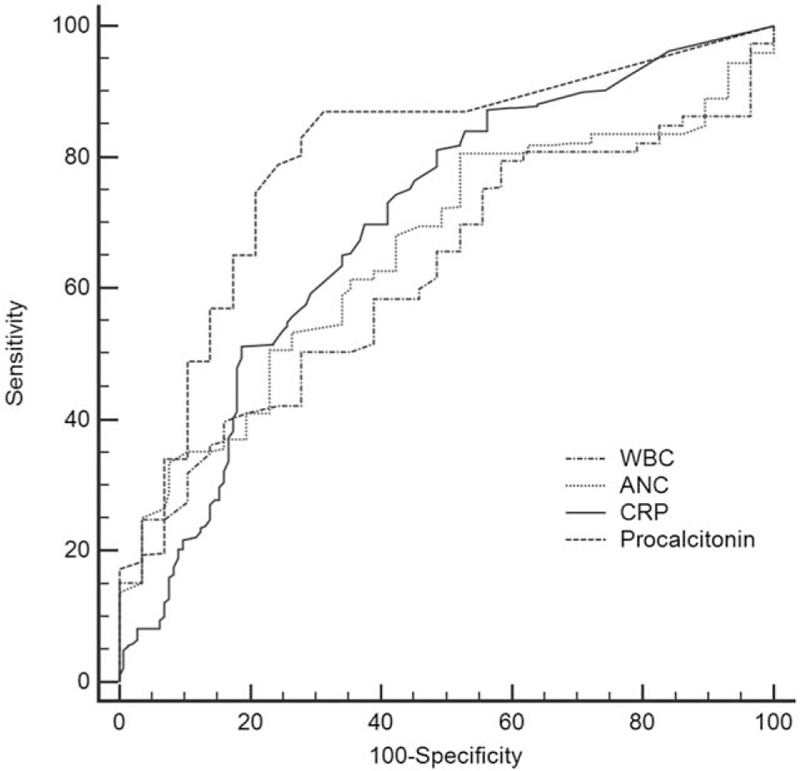
ROC analysis to differentiate inflammatory from non-inflammatory diarrhea. ANC = absolute neutrophil count, CRP = C-reactive protein, ROC = receiver operator characteristic, WBC = white blood cells.

The level of procalcitonin was the main determinant of inflammatory diarrhea (odds ratio [OR] 1.321, *P* < .001). Procalcitonin had a high value of area-under-the-curve (AUC) of 0.797 (95% confidence interval (CI) [0.760, 0.831]; *P* < .001) within the ROC diagnosis to differentiate non-inflammatory from inflammatory diarrhea (Table 4 and Fig. 2). In the inflammatory diarrhea analysis, procalcitonin had a sensitivity of 87.03% and a specificity of 68.75% at a cut-off level of 0.08 ng/mL. CRP also had a comparatively high AUC value of 0.697 (95% CI [0.656, 0.737]; *P* < .001), although its sensitivity (81.08%) and specificity (51.39%) were less than procalcitonin.

**Table 4:**
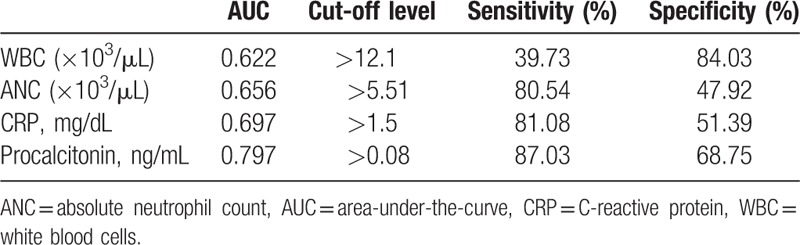
ROC analysis to differentiate inflammatory from non-inflammatory diarrhea with diverse serum indicators of infection.

## 4 Discussion

As confirmed in our study, clinical symptoms cannot be used to dependably differentiate non-inflammatory diarrhea from inflammatory diarrhea in patients with acute infectious diarrhea. In this retrospective study, we separated patients with acute infectious diarrhea into 2 groups, non-inflammatory and inflammatory diarrhea, based on the results from an abdominal CT or colonoscopy. We compared the clinical characteristics of the 2 groups and explored the aptitude of various inflammatory indicators in differentiating between them. We found that the procalcitonin levels in patients with acute infectious diarrhea could help clinicians differentiate between non-inflammatory and inflammatory diarrhea. Currently, little research has been done on the precision of using procalcitonin to differentiate between non-inflammatory and inflammatory diarrhea. In our study, which involved 514 patients, we demonstrated that the determination of serum procalcitonin could have significant predictive value (OR 1.321, AUC 0.797) for the analysis of inflammatory diarrhea, and offered a better predictive value compared with CRP (OR 1.145, AUC 0.697). In the inflammatory diarrhea analysis, procalcitonin had a sensitivity of 87.03% and a specificity of 68.75% at a cut-off level of 0.08 ng/mL.

There are 2 studies about the usefulness of measuring procalcitonin levels from a prospective setting. In a study performed by Herrlinger et al,^[21]^ patients with self-limited enterocolitis showed significantly higher procalcitonin levels when compared with inflammatory bowel disease patients (0.36 ng/mL, 95% CI [0.18, 1.7] vs 0.10 ng/mL, 95% CI [0.08, 0.5]; *P* < .001). Using the cut-off level procalcitonin ≥0.4 ng/mL, the sensitivity for self-limited colitis was 92% and the specificity was 96%. The positive predictive value for self-limited colitis was 96%, whereas the negative predictive value was 93%. Thia et al^[22]^ evaluated the utility of procalcitonin in diagnosing gastroenteritis. Using the cut-off level procalcitonin ≥0.5 ng/mL, the sensitivity for bacterial gastroenteritis was 40% and the specificity was 92%. When a lower procalcitonin ≥0.1 ng/mL cut-off level was chosen, the sensitivity was higher (93%) but the specificity was reduced to 50%. Based on the AUC for the ROC curve, procalcitonin performed well in the prediction of bacterial gastroenteritis, with an AUC of 0.727 (95% CI [0.580, 0.874]; *P* = .006).

In the observational studies, the probability of bacterial infection was defined as very unlikely (<0.1 ng/mL), unlikely (0.1–0.25 ng/mL), likely (0.25–0.5 ng/mL), and very likely (>0.5 ng/mL) according to the cut-off levels of procalcitonin. The use of antibiotics was recommended when the procalcitonin level was higher than 0.25 ng/mL, while avoiding the use of unnecessary antibiotics when the procalcitonin level was lower than 0.25 ng/mL.^[23]^ Some authors have shown the importance and role of procalcitonin in randomized controlled trials rather than observational studies.^[24,25]^ Ismaili-Jaha et al^[26]^ classified the etiology of diarrhea according to the cut-off levels of procalcitonin in patients with diarrhea. First, the mean and peak values of procalcitonin in patients with diarrhea due to viral infection were 0.133 and 2.30 ng/mL, respectively. Second, the mean and peak values of procalcitonin in patients with diarrhea due to bacterial infection were 5.30 and 18.0 ng/mL, respectively. Finally, the mean and peak values of procalcitonin in patients with extra-intestinal diarrhea (sepsis, meningitis) were 1.658 and 12.40 ng/mL, respectively.

The main method of detecting bacterial infection is through culture. Examinations to identify viral infections involve acute and convalescent-stage antibody titers and trials for viral antigens. However, outcomes using these methods can be delayed, while fast immunological or genomic assessments require information about the infectious agent. Early detection of bacterial infections could potentially direct management and decrease the abuse of antibiotics, causing enhanced long-term outcomes.^[27]^ Among numerous indicators of inflammation and sepsis, procalcitonin, an acute-stage reactant, has been researched for its capability to precisely detect bacterial infection.

During systemic inflammation, specifically in bacterial infections, under the impact of inflammatory cytokines and bacterial endotoxins, procalcitonin, which is synthesized in several tissues, such as the lung, kidney, liver, and adipose tissue, enters the circulatory system. At this stage, the level of procalcitonin can increase up to 1000 times that of the original level.^[29,30]^ The initial detectable values of procalcitonin are established 2 to 4 hours following stimulation; the highest levels of procalcitonin are reached within 6 to 24 hours.^[28,29]^ In contrast, the CRP level increases 12 to 24 hours following stimulation, attaining the highest level after 48 hours.^[28]^ The concentration of procalcitonin is not influenced by neutropenia, immunodeficiency circumstances, or the use of nonsteroidal and steroidal anti-inflammatory treatments, which is not the situation with CRP.^[30]^ As an increase in the level of procalcitonin corresponds to the intensity of the inflammatory response and the severity of the infection, thus, increased concentrations or continuously high levels can be prognostic markers for severe types of infection with adverse outcomes.^[31]^ Based on the technique of measurement used, the procalcitonin level within a patient's blood can be measured within a range of 19 minutes to 2.5 hours.^[28,29]^ Circulating procalcitonin level is halved in 24 hours after treatment of the infection, either by the immune system or by an antibiotic,^[32]^ which makes it an indicator of the usefulness of the treatment. In addition, studies have demonstrated that the addition of procalcitonin in treatment guidelines decreases the use of antibiotics without adverse consequences affecting the final outcome of the infection.^[33,34]^

The American College of Gastroenterology recommends the use of empirical antibiotic therapy in adult gastroenteritis patients with acute infectious diarrhea.^[35]^ Therefore, it is essential to recognize patients that have acute infectious diarrhea caused by bacteria. Thia et al^[22]^ found that detecting the level of procalcitonin helped in the discrimination of bacterial and undifferentiated gastroenteritis. However, they used stool cultures that were not sensitive enough to diagnose bacterial gastroenteritis. Our study employed much more sensitive means (colonoscopy and imaging research). Moreover, our study included a larger study population compared with that of Thia et al.

There are 2 new knowledges from this study. First, this is the rare study of serum procalcitonin levels in differentiating between inflammatory and non-inflammatory diarrhea in patients with acute infectious diarrhea. Currently, to our knowledge, there are very few reports about the usefulness of serum procalcitonin in acute infectious diarrhea. Second, in the inflammatory diarrhea analysis, procalcitonin showed superior sensitivity and specificity compared with CRP. The serum procalcitonin levels can be measured quickly and it helps to make an early diagnosis in an emergent situation such as systemic inflammation or septic shock. Inflammatory diarrhea can have serious complications if not treated with antibiotics especially in old age and immunocompromised patients. On the other hand, treating non-inflammatory diarrhea with antibiotics is not only unsuccessful, but also increases antibiotic resistance, costs of treatment, and toxicity of drug and allergic reactions. Therefore, the level of procalcitonin in a patient with acute infectious diarrhea on admission might assist with clinician's decision-making, such as whether to begin empirical antibiotic treatment.

However, our study has some limitations. First, it was a retrospective study. This means that, the patient information might be inaccurate. Second, since all patients with acute infectious diarrhea included in this study were diagnosed and treated at our single center, there are restrictions regarding general representability because of a relatively small sample size. Third, inflammatory and non-inflammatory diarrhea patients were differentiated by colonoscopy and imaging research; no microscopic stool examinations or stool cultures were conducted. Fourth, we only measured procalcitonin levels on admission, and did not follow procalcitonin levels, because we wanted to see if a single measurement on admission can differentiate between inflammatory and non-inflammatory diarrhea. Fifth, there were insufficient investigations of other co-morbidities except hypertension and diabetes. Patients with cancer or another immunocompromised disease are more susceptible to infection and may have higher procalcitonin levels. Finally, it is a relatively low specificity value of procalcitonin. Because the medical insurance cost of national health insurance corporation is relatively low, a large number of patients visit tertiary hospitals despite of mild symptoms. This high health service accessibility resulted in high percentage of patients who draw blood sample early before the procalcitonin levels rise sufficiently. As a result, the cut-off level is determined to be low and the specificity is decreased.

It is important to realize that at this point, procalcitonin cannot be suggested as a gold standard diagnostic method for inflammatory diarrhea, although, it should be considered in conjunction with other medical, diagnostic, and/or microbiological information. Given the limitations of procalcitonin as a solitary biomarker, additional large-scale prospective studies should be performed to determine its diagnostic value.

In conclusion, our study reveals that procalcitonin is a good candidate biomarker in differentiating between inflammatory and non-inflammatory diarrhea in patients with acute infectious diarrhea.

## Author contributions

**Conceptualization:** Hae Jin Shin, Sun Hyung Kang, Jae Kyu Sung, Seok Hyun Kim.

**Data curation:** Hae Jin Shin, Sun Hyung Kang, Jae Kyu Sung, Seok Hyun Kim.

**Formal analysis:** Hae Jin Shin, Sun Hyung Kang, Byung Seok Lee.

**Investigation:** Hae Jin Shin, Hee Seok Moon, Eaum Seok Lee.

**Methodology:** Hae Jin Shin, Sun Hyung Kang, Hee Seok Moon, Hyun Yong Jeong, Eaum Seok Lee.

**Project administration:** Hae Jin Shin, Sun Hyung Kang, Byung Seok Lee.

**Resources:** Hee Seok Moon, Ju Seok Kim.

**Software:** Hae Jin Shin, Jae Kyu Sung, Jong Seok Joo.

**Supervision:** Sun Hyung Kang, Hyun Yong Jeong, Byung Seok Lee.

**Validation:** Hae Jin Shin, Sun Hyung Kang, Hyun Yong Jeong.

**Visualization:** Hae Jin Shin, Sun Hyung Kang, Ju Seok Kim, Jong Seok Joo.

**Writing – original draft:** Hae Jin Shin.

**Writing – review and editing:** Hae Jin Shin, Sun Hyung Kang.
